# RNA-seq analysis reveals different drought tolerance mechanisms in two broadly adapted wheat cultivars ‘TAM 111’ and ‘TAM 112’

**DOI:** 10.1038/s41598-021-83372-0

**Published:** 2021-02-22

**Authors:** Chenggen Chu, Shichen Wang, Li Paetzold, Zhen Wang, Kele Hui, Jackie C. Rudd, Qingwu Xue, Amir M. H. Ibrahim, Richard Metz, Charles D. Johnson, Charles M. Rush, Shuyu Liu

**Affiliations:** 1 Texas A&M AgriLife Research Center, 6500 Amarillo Blvd W, Amarillo, TX 79106 USA; 2 Genomics and Bioinformatics Service Center, Texas A&M AgriLife Research, College Station, TX 77843 USA; 3 Soil and Crop Sciences Department, Texas A&M University, College Station, TX 77843 USA; 4 Sugarbeet and Potato Research Unit, Edward T. Schafer Agricultural Research Center, USDA-ARS, 1616 Albrecht Blvd. N, Fargo, ND 58102 USA

**Keywords:** Biotechnology, Genetics

## Abstract

Wheat cultivars ‘TAM 111’ and ‘TAM 112’ have been dominantly grown in the Southern U.S. Great Plains for many years due to their high yield and drought tolerance. To identify the molecular basis and genetic control of drought tolerance in these two landmark cultivars, RNA-seq analysis was conducted to compare gene expression difference in flag leaves under fully irrigated (wet) and water deficient (dry) conditions. A total of 2254 genes showed significantly altered expression patterns under dry and wet conditions in the two cultivars. TAM 111 had 593 and 1532 dry–wet differentially expressed genes (DEGs), and TAM 112 had 777 and 1670 at heading and grain-filling stages, respectively. The two cultivars have 1214 (53.9%) dry–wet DEGs in common, which agreed with their excellent adaption to drought, but 438 and 602 dry–wet DEGs were respectively shown only in TAM 111 and TAM 112 suggested that each has a specific mechanism to cope with drought. Annotations of all 2254 genes showed 1855 have functions related to biosynthesis, stress responses, defense responses, transcription factors and cellular components related to ion or protein transportation and signal transduction. Comparing hierarchical structure of biological processes, molecule functions and cellular components revealed the significant regulation differences between TAM 111 and TAM 112, particularly for genes of phosphorylation and adenyl ribonucleotide binding, and proteins located in nucleus and plasma membrane. TAM 112 showed more active than TAM 111 in response to drought and carried more specific genes with most of them were up-regulated in responses to stresses of water deprivation, heat and oxidative, ABA-induced signal pathway and transcription regulation. In addition, 258 genes encoding predicted uncharacterized proteins and 141 unannotated genes with no similar sequences identified in the databases may represent novel genes related to drought response in TAM 111 or TAM 112. This research thus revealed different drought-tolerance mechanisms in TAM 111 and TAM 112 and identified useful drought tolerance genes for wheat adaption. Data of gene sequence and expression regulation from this study also provided useful information of annotating novel genes associated with drought tolerance in the wheat genome.

## Introduction

Drought (water deficit stress) is a major environmental stress threatening wheat (*Triticum aestivum* L.) productivity worldwide particularly under modeled global climate change scenarios. It is estimated that almost 50% of wheat cultivated in the developing world is sown under dryland cropping systems^1^. Increasing resilience to abiotic stresses through genetic improvement thus become a critical component of wheat breeding. To identify genes related to drought tolerance and understand mechanisms of plant reaction to water deficit in this type of germplasm is crucial for developing cultivars with better water use efficiency.

Drought can affect wheat growth during all phenological stages but heading and grain filling are two sensitive stages^2^ since they involve processes such as fertilization, embryogenesis, photosynthesis and starch biosynthesis during seed development. All these processes affect yield components of seed number per spike and kernel weight. Plants may avoid drought by flowering earlier and/or decreasing growth duration, or by reducing canopy size and regulating stomatal openings to enhance water uptake efficiency^3^. Furthermore, cellular osmosis adjustment^4^, antioxidant defense or oxidation-reduction^5^, stay-green character^6^, reserve translocation^7^ and hormonal regulations^8^ are among mechanisms plants use to cope with water deficit stress. Meanwhile cellular and metabolic processes are changed to initiate a series of regulatory networks for modulating drought tolerance^9^ such as differential expression of cytochrome, heat shock proteins, dehydrins, glutathione transferase, proteinase inhibitors, and regulatory proteins including transcription factors^10–13^. Plant responses to drought thus are affected by various factors including growth conditions, physiology and genotype, and they involve diverse gene expression patterns and signal pathways^14^, indicating that different genotypes would have specific mechanisms in dealing with water deficit stress. To efficiently utilize drought-tolerant germplasms in breeding, it is essential to uncover their genetic mechanism and identify unique drought response genes from different sources, which will further lead to development of cultivars with enhanced drought tolerance.

The Southern Great Plains (SGP) of the U.S. include the major winter wheat growing areas in Nebraska, Kansas, Oklahoma and Texas, and has a semi-arid climate with annual precipitation averaging about 480 mm and wheat growing season precipitation around 250 mm^15^. However, the seasonal evapotranspiration for winter wheat growth ranges from 700 to 950 mm for maximum grain yield under full irrigation conditions^15^. This shows that the seasonal precipitation for winter wheat in SGP only meets one-third of the evapotranspiration requirement. Therefore, development of drought tolerant cultivars with improved water-use efficiency will greatly enhance wheat yield in this area. Two winter wheat cultivars, ‘TAM 111’ and ‘TAM 112’, developed by Texas A&M AgriLife Research and released in 2003 and 2005, respectively^16,17^, showed excellent drought tolerance with consistently high yields under both dryland and irrigated conditions, and continuously ranked high in SGP for many years (Texas wheat variety survey, https://varietytesting.tamu.edu/wheat/). The two cultivars are also used in crossing blocks of U.S and global breeding programs for improving drought tolerance. Reddy et al.^18^ used microarray analysis to compare gene expression in TAM 111 and TAM 112 under water deficit stress and fully irrigated condition, and identified 1657 transcripts commonly existing in both cultivars that showed expression difference under the two conditions with transcripts mostly related to photosynthesis, carbohydrate metabolism, phytohormone metabolism, and other dehydration responses. However, the microarray used only a subset of genes and thus limited ability of detecting drought-response genes beyond the set. In addition, Xue et al.^19^ indicated that TAM 111 and TAM 112 showed different water-use efficiency and stem dry weight at anthesis for response to drought, indicating the two cultivars might have different mechanisms of drought tolerance. Therefore, identifying and comparing the genome-wide response to water deficit stress between TAM 111 and TAM 112 will uncover drought tolerance mechanisms, and aid the breeding processes by determining the key pathways.

By using high-throughput sequencing technologies, RNA-Seq can quantify gene expression levels with high accuracy in the entire transcriptome^20^ and reveal the precise location of transcription boundaries as well as sequence variations^21^. Recently, RNA-Seq has been successfully utilized in wheat for analyzing expression of genes response for heat stress^13,22^, salt stress^23,24^, drought^22,25,26^ and flowering regulation^27^. Particularly, with the availability of whole genome sequences in wheat landrace ‘Chinese Spring’^28^ and pan-genome sequences (www.10wheatgenomes.com) from ten wheat cultivars, physical positions of genes identified through RNA-seq can be easily determined. Therefore, using RNA-Seq analysis can not only detect differentially expressed genes (DEGs) as candidate genes for the target traits, but also verify genes/QTLs identified through genetic linkage and association analysis according to corresponding genomic positions. Additionally, gene sequence from RNA-Seq can be used to develop genic markers for marker-assisted selection and genomic selection in drought tolerance breeding^29^.

This research aims to identify drought responsive genes in TAM 111 and TAM 112. The two cultivars were grown in a greenhouse under fully irrigated (wet) and drought (dry) conditions, and flag leaves from main tillers of the two cultivars were collected at heading and grain filling stages for RNA-seq studies. By comparing differentially expressed genes (DEGs) between the two cultivars under the irrigated and drought conditions, we identified the gene regulatory networks that are common between the two cultivars, as well as those specific to each.

## Materials and methods

### Plant materials and water deficit treatment

The two hard red winter bread wheat cultivars, TAM 111 and TAM 112, have excellent drought tolerance and were developed by Texas A&M AgriLife Research^16,17^. TAM 111 has the pedigree ‘TAM 107’//TX78V3620/CTK78/3/ TX87V1233, and TAM 107 has the pedigree ‘TAM 105’*4/’Amigo’. It had higher grain yield in irrigated conditions but lower grain yield under extended drought conditions than that of TAM 112. TAM 112 was derived from the cross U1254-7-9-2-1/TXGH10440 where the male parent was a TAM 110 sib (has pedigree ‘TAM 105’*4/’Amigo’)*5//’Largo) and TAM 107 sib was in the recurrent parent^30^. TAM 200 (has pedigree 391-56-D8/’Tascosa’//’Centurk’) *3/’Amigo’) was in the pedigree of the female parent U1254-7-9-2^17^.Therefore, the two cultivars are related with partially shared pedigree about less than 40% in common. TAM 112 is 2–3 days earlier on heading and maturity than TAM 111 but with similar height because both have *Rht1*. TAM 112 has 1AL.1RS, *Gb3* for greenbug resistance to biotype E, and *Cmc*_*TAM112*_ for resistance to wheat curl mite and is the most drought tolerant winter wheat in the US Great Plains^19,31–33^.

The water deficit treatments and sample collection were as described in Reddy et al.^18^. Briefly, a randomized complete block design (RCBD) was used with the sampling time (heading, HD and grain filling, GF) as blocks. Three replicates were included in each block, and four pots within each replicate were used for each treatment with one pot containing three plants of one cultivar, which resulted in the total of 16 pots (48 plants) per replication and 48 pots (144 plants) in each block. The pots were maintained at 100% gravimetric water content (GWC) for the first seven weeks. Water deficit treatments were started at the jointing stage (50 days after transplanting) with GWC reduced to around 50% for the dry treatment but maintained at 90% for the wet treatment.

### RNA extraction, library construction and sequencing

Wheat flag leaves are the main organs for implementing photosynthesis and providing the major assimilate source for plant growth, spike development and sensing environmental signals for adaptation^34^. Wheat flag leaf can contribute as high as 45–58% of photosynthetic performance under favorable conditions^35^ with 41–43% of assimilates used in grain filling after flowering^36^. Therefore, flag leaf tissues from primary tillers of four individual plants in each replicate were collected and pooled as one sample at each stage of heading (HD) (79 days after transplanting) and middle grain filling (GF) (100 days after transplanting, 21 days after heading at Feekes 11.1 milky stage). Leaf samples were immediately put into liquid nitrogen after their harvest and then stored at − 80 °C for processing. RNA was extracted using a Qiagen-RNeasy Mini kit according to instruction provided by the manufacturer. For preparing sequencing libraries, the TruSeq RNA Sample Preparation kit v2 was used following the manufacturer’s instructions. Briefly, mRNA was purified by using poly-T oligo-attached magnetic beads, cDNA was then synthesized after fragmentation, followed by A-tailing, adaptors ligation and PCR amplification. After quality check, each library was then deeply sequenced on a single lane of Illumina HiSeq 2000 to generate 75 bp pair-end reads.

### Transcriptome and gene expression level analysis

Transcriptome and gene expression analysis were conducted using computer program Cufflinks according to Trapnell^37^. Raw reads were filtered by removing adapters and trimming low quality bases (Phred score < 20) at the end of reads using Trimmomatic v0.38^38^. The wheat genome RefSeq v1.0 assembly and gene model annotation files were downloaded from the International Wheat Genome Sequencing Consortium (IWGSC)^28^ (https://urgi.versailles.inra.fr/download/iwgsc/IWGSC_RefSeq_Assemblies/v1.0/). The filtered reads were then aligned to the reference genome using tool TopHat2^39^. Transcript assembles were generated using the computer package Cufflinks^37^, and all assembled transcripts were further merged through the tool Cuffmerge using the wheat reference gene annotation as a guide. The tool Cuffdiff that was included in the Cufflinks package was then used to calculate gene expression level and normalize transcripts abundance to FPKM value (Fragment per kilobase of transcript per million reads mapped) and then make calls of differentially expressed genes (DEGs). The significance (*P* values) of DEGs were adjusted using the Benjamini and Hochberg multiple test correction approach^40^ to control the false discovery rate (FDR), and only the genes with an adjusted *P* value < 0.05 (FDR < 0.05) and had a fold change value greater than two (the absolute value of Log2 [expression level under dry/wet] is no less than one) were considered to have significant expression difference. In this research, the gene expression comparison was performed separately between two treatments (dry vs. wet) and between two cultivars (TAM 111 vs. TAM 112). In addition, the gene expression regulation was considered as up-regulated if the expression level is higher under the dry conditions (Log2[fold change] > 0), and down-regulated if the expression level is lower under drought treatment (Log2[fold change] < 0).

### Gene functional assignment

The computer program Blast2Go^41,42^ was used for functional annotation of the genes with significantly different expression levels under dry and wet conditions. The tool NCBI BLAST was used to compare DNA sequence of each DEGs with those stored in NCBI GenBank database (https://www.ncbi.nlm.nih.gov/) to infer gene function with BLAST expectation value (E value) threshold set at 1.0 × 10^–5^, and the tool InterProscan using the inferred protein sequences (motifs) to search against InterPro databases with the web service provided by EMBL-EBI (https://www.ebi.ac.uk/interpro/), to find gene ortholog (GO) related information. The tool GO Mapping was used to retrieve GO terms associated with BLAST search hits from NCBI GO database and online protein databases (such as PSD, UniProt, SwissProt, TrEMBL, RefSeq, GenPept and PDB). The tool Gene Ontology Annotation was then used to assign GO terms to gene sequences, and tool Gene Ontology Graphs was used to develop hierarchical structure of the gene ontology according to biological processes, protein molecule functions and subcellular localization of the proteins. For genes with ambiguous function descriptions from Blast2Go annotation, gene sequences were manually searched against NCBI^43^, and the translated protein sequences were used to search against online protein databases again to infer their function.

### Comparing TAM 111 and TAM 112 gene expressions responding to drought

To understand drought tolerance mechanisms in TAM 111 and TAM 112, comparison of gene expression difference between dry and wet conditions in each cultivar and expression difference between them under dry or wet conditions were separately conducted. DEGs common in both cultivars thus represented the similar mechanisms they shared for response to drought, and DEGs specific to each cultivar or showing different regulation patterns between them were the indication of unique drought-tolerance mechanism in each. The annotated genes response to drought were then used to develop hierarchical diagrams according to their roles in biological processes, molecule functions and cellular component locations through Blast2Go^41,42^, and the graphs of each cultivar were used to find the difference between TAM 111 and TAM 112 for their drought response. In addition, expression comparison of genes in the process of responding to water deficit stress, other drought related processes of heat and oxidative stress response, abscisic acid (ABA)-activated signal pathway and transcription regulations were also performed in the two cultivars to determine their specificity in drought tolerance.

### RT-qPCR analysis

To confirm the RNA-seq results, the expression levels of four differentially expressed genes (two were up-regulated and two were down-regulated) were analyzed through real-time quantitative polymerase chain reaction (RT-qPCR) using an Applied Biosystems ViiA 7 Real-Time PCR System. Reverse transcription was performed using Qiagen’s Omniscript RT Kit (Cat. # 205113) via standard protocol described by manufacturer. Primers of four genes were designed using Primer 3 web version 4.1.0 (https://bioinfo.ut.ee/primer3/) and then BLAST search against wheat reference genomic sequence (https://wheat-urgi.versailles.inra.fr/Seq-Repository/BLAST) to check specificity of the primers. Expressions of four genes were determined using Comparative Quantification and (ΔΔCt) method using Actin gene as an endogenous control. PCR amplification was conducted in a 20 µl mix contained 20 ng of cDNA template, 1 × SYBR Select Master Mix (Applied Biosystem), and 0.3 µM each of forward and reverse primers. Thermal cycling conditions were set at 50 °C for 2 min, 95 °C for 10 min, followed by 40 cycles of denaturing at 95 °C for 15 s and annealing at 60 °C for 1 min, with fluorescence detection at the end of each cycle. The amplification of a single product per reaction was confirmed by melting curve analysis. The primers sequences of four genes and the endogenous control (Actin gene) were listed in Table S1.

## Results

### Sequencing summary

In total, we generated > 720 Million 75 bp pair-end reads, which is about 47 Million reads per sample. After quality trimming and filtering, 671 Million reads were retained, yielding an average of 44 Million reads per sample. The average GC percentage is 51.7%, and the Q30 percentage is 95.3%. When mapping the RNA-seq reads to the reference genome, ~ 87.01% of the quality-filtered reads could be mapped and among these mapped reads, ~ 84.87% could be mapped uniquely to single location.

### Overall expression profile in TAM 111 and TAM 112

**Figure 1:**
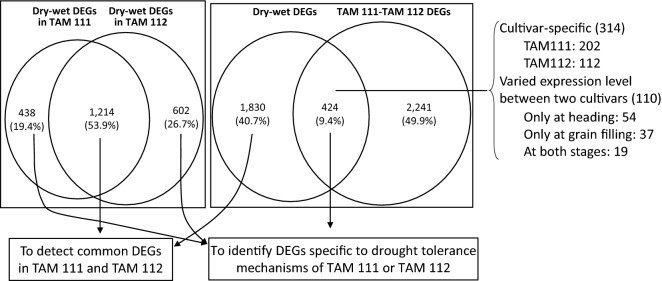
Differentially expressed genes (DEGs) revealed by expression comparison between dry and wet conditions in TAM 111 and TAM 112 and between TAM 111 and TAM 112 wheats under dry and wet conditions.

From sequencing reads of three replicates in TAM 111 and TAM 112 under dry and wet conditions at heading and grain filling stages, 122,017 sequences with length greater than 80 bp were assembled and mapped to wheat genome according to the IWGSC RefSeq v1.0^28^. Of those genes, 2254 showed significantly altered expression levels between dry and wet treatments in one of the two cultivars. In total, TAM 111 had 1652 and TAM112 had 1816 dry–wet DEGs with 1214 (53.9%) being common in both cultivars, and 438 (19.4%) and 602 (26.7%) specific to TAM 111 and TAM 112, respectively (Fig. 1). TAM 111 had 593 dry–wet DEGs at heading and 1532 at grain filling, and TAM 112 had 777 at heading and 1670 at grain-filling (Table 1). Overall, TAM 112 had more dry–wet DEGs detected than those of TAM 111 for both heading and grain filling stages. However, 53.9% of the dry–wet DEGs were common in both cultivars, suggesting that many of the drought response pathways are conserved between the two cultivars. In addition, some dry–wet DEGs also showed specificity to growth stage. For example, of the 1214 common in the two cultivars, 25 and 520 dry–wet DEGs were significant only at stages of heading and grain filling, respectively (Table 2), which implied that some responses are growth stage specific.

**Table 1:** Comparison of differentially expressed genes (DEGs) between dry and wet conditions within and between TAM 111 and TAM 112 at heading (HD) and grain filling (GF) stages.

Comparison	DEGs
Between dry and wet treatments	2254
TAM 111	1652
TAM 111 at HD	593
TAM 111 at GF	1532
TAM 112	1816
TAM 112 at HD	777
TAM 112 at GF	1670
Between TAM 111 and TAM 112	2665
Dry	1781
Dry at HD	647
Dry at GF	1541
Wet	1865
Wet at HD	1621
Wet at GF	650

**Table 2:** The dry–wet differentially expressed genes (DEGs) identified in wheat cultivars TAM 111 and TAM 112 at heading (HD) and grain filling (GF) stages.

Significant DEGs	TAM 111		TAM 112	
	HD	GF	HD	GF
**Common in two cultivars (1214 genes)**				
Both cultivars at both HD and GF	276	276	276	276
TAM111 at HD, TAM 112 at HD&GF	14	0	14	14
TAM111 at GF, TAM 112 at HD&GF	0	276	276	276
TAM111 at HD&GF, TAM 112 at HD	33	33	33	0
TAM111 at HD&GF, TAM 112 at GF	70	70	0	70
Both cultivars at HD only	25	0	25	0
Both cultivars at GF only	0	520	0	520
**Cultivar-specific (1040 genes)**				
TAM 111 at HD & GF	94	94	0	0
TAM 112 at HD & GF	0	0	65	65
TAM 111 at HD	81	0	0	0
TAM 111 at GF	0	263	0	0
TAM 112 at HD	0	0	88	0
TAM 112 at GF	0	0	0	449
Total (2254 genes)	593	1532	777	1670

Comparison of gene expression between TAM 111 and TAM 112 found 2665 genes to have significantly different expression levels with 1781 under drought and 1865 under well-watered conditions (Table 1). Interestingly, the number of dry–wet DEGs between two cultivars at the two growth stages varied according to the growth conditions. Under well-watered condition, more dry–wet DEGs between the cultivars were found at the heading (1621) than grain filling stage (650), whereas under the dry treatment, more dry–wet DEGs were found at the grain filling (1541) than the heading stage (647) (Table 1), indicating that the two cultivars had different strategies for responding to water stress at those stages. When DEGs of dry vs. wet and TAM 111 vs. TAM 112 were intersected, only 424 (9.4%) were found in common which included 314 that were cultivar specific and 110 that had significant fold change difference between the two cultivars for drought response (Fig. 1). These 424 dry–wet DEGs were considered the genes regulated specifically in either cultivar, and were further analyzed to understand specific drought tolerance mechanisms in each cultivar (Fig. 1). Likewise, the DEGs found in dry vs. wet comparison in both cultivars with similar fold change were identified as those regulated similarly between the two cultivars and were further analyzed to uncover the common mechanisms of drought response in TAM 111 and TAM 112 (Fig. 1).

### Gene ontology annotation and enrichment analysis of DEGs in TAM 111 and TAM 112 under dry and wet conditions at heading and grain filling stages

Of the 2254 DEGs identified in TAM 111 and TAM 112 under dry and wet conditions at heading and grain filling stages, gene ontology (GO) annotation and NCBI BLAST against the online DNA and protein databases inferred the potential function of 1855 genes whereas function of 399 genes remained unknown. The 399 genes with unknown functions included 258 encoded either predicted or uncharacterized proteins and 141 had no BLAST hits (no similar DNA sequence) in NCBI DNA database and no similar GO terms can be determined according to current protein databases. However, using those 141 genes with no BLAST hits as queries to BLAST against IWGSC^28^ (https://urgi.versailles.inra.fr/blast_iwgsc/) sequence database, all of them had high similarity to the corresponding sequences (Table S2), suggesting that those no-hits sequences likely represented novel genes in the wheat genome and were related to drought response, especially manifested in TAM 111 and TAM 112.

**Figure 2:**
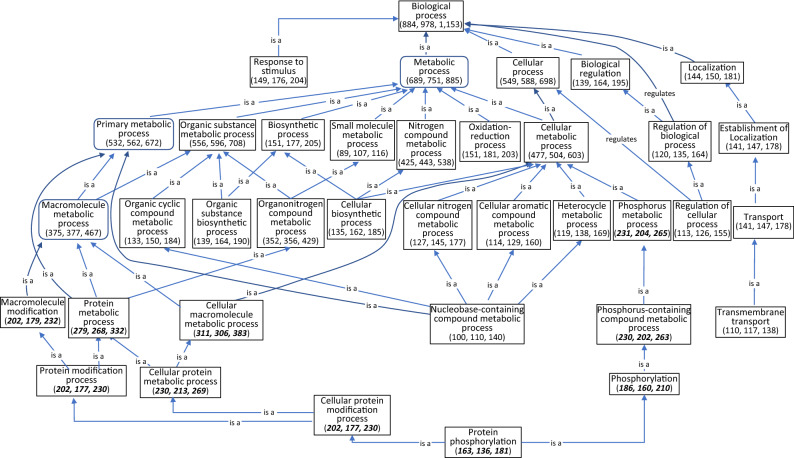
Hierarchical diagram of biological process according to 1153 annotated genes that were differentially expressed under dry and wet conditions in wheat cultivars TAM 111 and TAM 112. Numbers in parenthesis indicate the number of genes in each biological process with the first number indicating genes of TAM 111 followed by those of TAM 112 and the total number of unique genes in both cultivars combined. Numbers in bold and italic font style showed that TAM 111 had a higher number of DEGs for the corresponding biological process, which were different from the majority where TAM 112 had a higher number of DEGs.

According to 1855 genes with assigned functions, two sets of genes with significant dry–wet expression difference in TAM 111 (1389 genes) and TAM 112 (1549 genes) were used to develop hierarchical graphs in each cultivar according to their roles in biological processes, molecule functions and locations in cellular components. The hierarchical biological processes diagram included 1153 genes with 884 and 978 differentially expressed in TAM 111 and TAM 112, respectively (Fig. 2). For most of the biological processes, the number of genes associated with each process were greater in TAM 112 than TAM 111. However, the process of protein phosphorylation and other related biological processes such as protein modification process, cellular protein metabolic process, phosphorus and phosphorus metabolic process, etc., all showed more dry–wet DEGs in TAM 111 than TAM 112 (Fig. 2). In total, 181 dry–wet DEGs in TAM 111 and TAM 112 were involved in protein phosphorylation with 163 and 136 genes being differentially expressed only in TAM 111 and TAM 112, respectively (Fig. 2 and Table 3). Among them, 118 genes showed differential expression in both cultivars under dry and wet conditions with two at heading, 24 at grain filling and 80 at both stages. The expression data indicated that the majority (106 genes) of those common dry–wet DEGs were down regulated, and most of those are predicted to have a kinase activity. Of the 45 and 18 dry–wet DEGs involved in protein phosphorylation that were specifically found in TAM 111 and TAM 112, respectively, 43 TAM 111-DEGs were down-regulated whereas ten TAM 112-DEGs were up-regulated (Table 3). The functions of those down-regulated genes in TAM 111 were mostly related to kinase activity involved in defense response, signal transduction and programmed cell death etc. On the other hand, among ten up-regulated genes in TAM 112, three (L_066553, L_081188 and L_001137) were related to cell development, two (L_010317 and L_024447) were related to water deprivation stress response, one (L_005995) for photosynthesis and two (L_090655 and L_095893) for defense response (Table S3).

**Table 3:** Regulation of dry–wet differentially expressed genes (DEGs) involved in biological process, molecule functions and cellular components of TAM 111 and TAM 112 under water deficit stress at heading (HD) and grain filling (GF) stages.

Pathway function/Stages	Common in both cultivars		TAM 111-specific		TAM 112-specific	
	Down-regulated	Up-regulated	Down-regulated	Up-regulated	Down-regulated	Up-regulated
**Biological process—protein phosphorylation**						
Only at HD	2	0	9	1	3	0
Only at GF	24	5	30	1	5	10
At both HD and GF	80	7	4	0	0	0
**Molecule function—adenyl ribonucleotide binding**						
Only at HD	0	0	2	1	1	4
Only at GF	11	14	10	1	3	14
At both HD and GF	19	20	4	0	1	1
**Cellular component—nucleus**						
Only at HD	0	0	1	2	0	1
Only at GF	16	29	8	8	5	4
At both HD and GF	19	36	1	0	2	1
**Cellular component—plasma membrane**						
Only at HD	0	0	1	1	1	0
Only at GF	12	11	6	1	3	10
At both HD and GF	21	8	4	0	0	0

**Figure 3:**
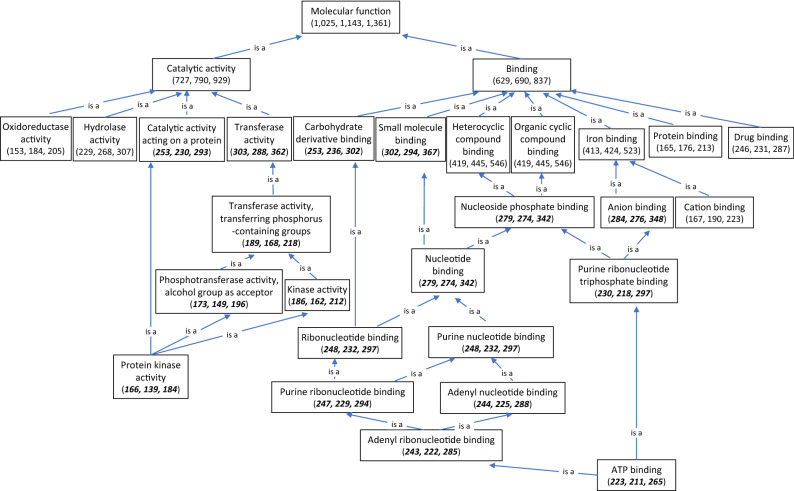
Hierarchical diagram of molecule function according to 1361 annotated genes that were differentially expressed under dry and wet conditions in TAM 111 and TAM 112. Numbers in parenthesis indicate the number of genes in each molecule function with the first number indicating genes of TAM 111 followed by that of TAM 112 and the total number of the unique genes in both cultivars combined. Numbers in bold and italic font style showed that TAM 111 had a higher number of DEGs for the corresponding molecule function, which were different from the majority where TAM 112 had a higher number of DEGs.

The hierarchical diagram of molecule functions was built based on 1361 genes with known functions, and 1025 and 1143 genes were differentially expressed in TAM 111 and TAM 112, respectively (Fig. 3). Similar to what was shown in the hierarchical biological process diagram, number of molecules with protein kinase activity showed significant difference between TAM 111 and TAM 112 with almost the same set of genes with protein phosphorylation functions. Molecules with the function of adenyl ribonucleotide binding also have more genes differentially expressed in TAM 111 than TAM 112 (Fig. 3). Of the total of 285 dry–wet DEGs having function of adenyl ribonucleotide binding, 243 and 222 were differentially expressed in TAM 111 and TAM 112, respectively. After removing genes overlapping with the sets involved in protein phosphorylation, 106 genes solely assigned to adenyl ribonucleotide binding remained with 64 commonly shown in both cultivars and 18 and 24 were specific to TAM 111 and TAM 112, respectively (Table 3). Of the 64 common dry–wet DEGs in two cultivars, 30 were down-regulated and 34 were up-regulated, but for DEGs that were cultivar specific, 16 out of 18 dry–wet DEGS in TAM 111 were down-regulated, while 19 out of 24 dry–wet DEGs in TAM 112 were up-regulated. Among common dry–wet DEGs of adenyl ribonucleotide binding in TAM 111 and TAM 112, down-regulated genes were mostly predicted to function in protein phosphorylation with kinase activity while 34 genes up-regulated were mostly involved in processes response to water deprivation, heat and oxidative stresses and transmembrane transporting (Table S4). As for specific dry–wet DEGs of adenyl ribonucleotide binding genes in each cultivar, down-regulated genes in TAM 111 were mostly related to defense response, whereas the up-regulated genes in TAM 112 were mostly involved in response to water deprivation, heat and oxidative stresses, ABA-signaling pathway and transmembrane transporting (Table S4).

**Figure 4:**
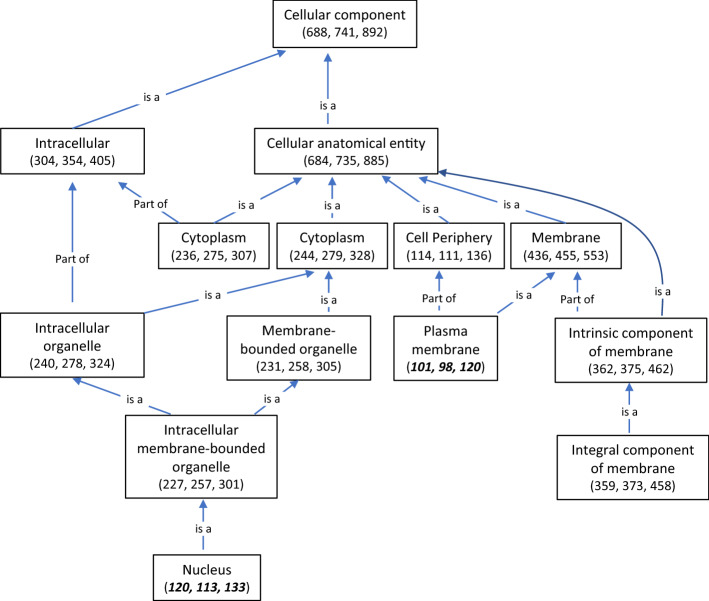
Hierarchical diagram of cellular components according to 892 annotated genes that were differentially expressed under dry and wet treatments in TAM 111 and TAM 112. Numbers in parenthesis indicate the number of genes in each cellular component with the first number indicating the genes in TAM 111 followed by that of TAM 112 and the total number in both cultivars. Numbers in bold and italic font style showed that TAM 111 had a higher number of DEGs for the corresponding cellular component, which were different from the majority where TAM 112 had a higher number of DEGs.

Among 892 genes with known functions used in developing hierarchical diagram according to protein locations in cellular components, 688 and 741 genes were differentially expressed in TAM 111 and TAM 112, respectively, with those localized in nucleus and plasma membrane showing significant differences between two cultivars (Fig. 4). A total of 133 genes encoded products predicted to localize in nucleus with 120 and 113 showed significant dry–wet differential expression in TAM 111 and TAM 112, respectively (Fig. 4 and Table 3), with 100 common in both cultivars, and 20 and 13 specific in TAM 111 and TAM 112, respectively (Table S5). Among the common dry–wet DEGs, 35 genes were down-regulated and 65 were up-regulated, whereas among dry–wet DEGs specifically shown in each cultivar, about half of them were up-regulated (Table 3). In TAM 111, the up-regulated genes were mainly involved in response to stresses and transcription regulating, whereas in TAM 112, those up-regulated genes were encoding chloroplastic proteins or were related to transmembrane activities (Table S5).

A total of 120 genes with encoded proteins predicted to localize in plasma membrane and showing expression difference were identified in the two cultivars (Fig. 4). Of those, 101 and 98 dry–wet DEGs were found in TAM 111 and TAM 112, respectively, which included 52 in common, 13 in TAM 111 and 14 in TAM 112 after excluding genes with products in nucleus membrane that overlapped with those listed in Table 3. The common dry–wet DEGs had 33 down-regulated and 19 up-regulated, whereas in those specific to cultivars, 11 out of 13 in TAM 111 were down-regulated and 10 out of 14 in TAM 112 were up-regulated. The down-regulated genes in TAM 111 were related to transmembrane transporting water, amino acid and protein, or defense response, while the up-regulated genes in TAM 112 were involved in processes related to transmembrane transporting, metabolism and biosynthesis (Table S6). Based on comparison of hierarchical graphs developed in each cultivar, in addition to the common dry–wet DEGs that occupied a main portion of genes differentially expressed in two cultivars, significantly different gene regulations were observed in cultivar-specific dry–wet DEGs with more genes in TAM 112 being up-regulated under drought. This indicates that TAM 112 is relatively more active than TAM 111 in responding to drought.

### Expression of genes response to water deprivation stress in TAM 111 and TAM 112 under dry and wet conditions at heading and grain filling stages

**Figure 5:**
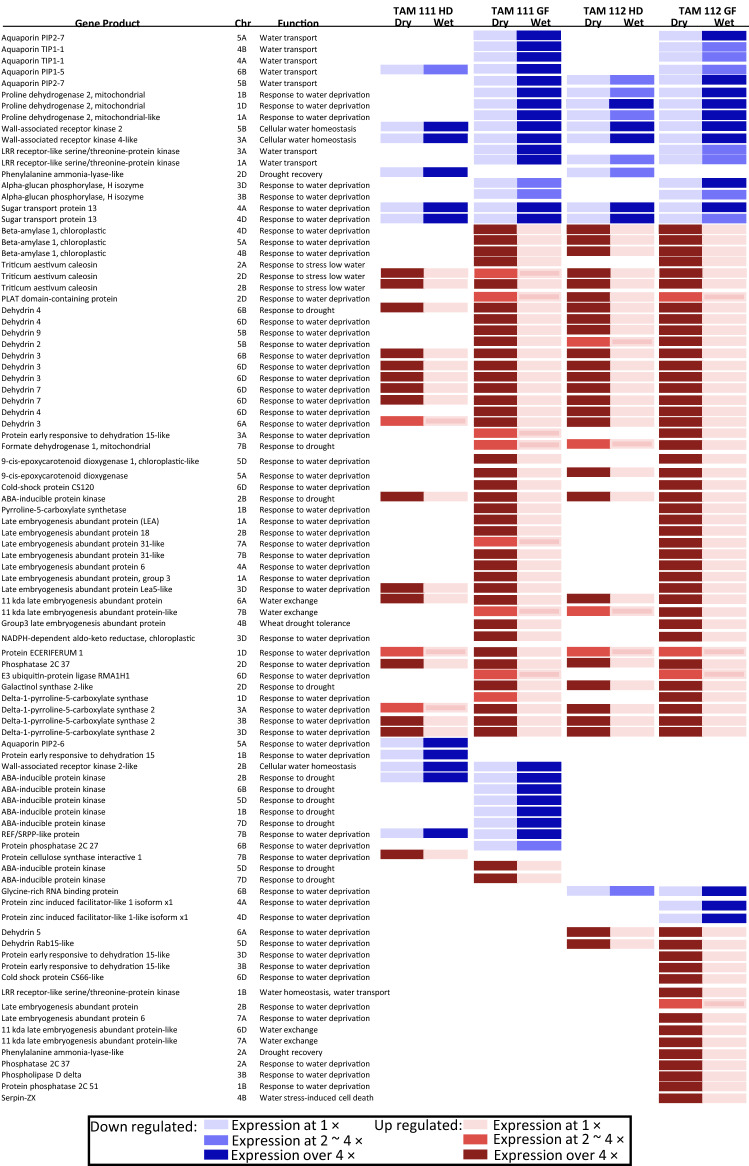
Expression heat map of differentially expressed genes (DEGs) response to water deprivation stress in TAM 111 and TAM 112 under dry and wet conditions at heading and grain filling stages. Chr = Chromosome, HD = heading stage, and GF = grain filling stage.

Among 1855 genes with assigned functions, a total of 92 genes involved in water deficit stress were differentially expressed in at least one of the two cultivars at either heading or grain filling stages, with 61 dry–wet DEGs being common in both cultivars, and 13 and 18 specific to TAM 111 and TAM 112, respectively (Fig. 5 and Table S7). The functions of the encoded proteins include amylase, aquaporin, caleosin, dehydrin, dioxygenase, heat/cold-shock protein, kinase, late embryogenesis abundant protein, oxidoreductase, phosphatase, synthase and transporters which were involved in processes of response to water deprivation, water transport and exchange and drought recovery (Table S7). Among 61 common dry–wet DEGs, 16 genes encoding aquaporin, phosphorylase, kinase, dehydrogenase and sugar transporter were down-regulated, and 44 genes encoding dehydrin, amylase, caleosin, late embryogenesis abundant protein and oxidoreductase were up-regulated under drought condition (Table S7). Among the 13 dry–wet DEGs specific to TAM 111, eleven genes encoding kinase, aquaporin, phosphatase synthase, dehydrogenase and lipid droplets-associated proteins were down regulated and two genes encoding ABA-inducible protein kinases were up regulated. On the other hand, for TAM 112-specific dry–wet DEGs, three genes encoding transcription factors and transmembrane transporter were down regulated and fifteen genes encoding dehydrin, dehydrogenase, heat shock protein, late embryogenesis abundant protein, lyase, phosphatase, protease and kinase were up-regulated (Fig. 5 and Table S7). Overall, the two cultivars shared over 60% of DEGs involved in response to water deprivation, which agrees with their excellent drought tolerance. However, based on cultivar-specific dry–wet DEGs, TAM 112 have more genes than TAM 111 for drought response with majority being up regulated, while the specific dry–wet DEGs in TAM 111 were mainly down regulated (Table S7), which was consistent with the comparison between two cultivars through hierarchy and further proves that TAM 112 was relatively more responsive to water deficit stress than TAM 111.

### Expression of genes response to heat and oxidative stresses, abscisic acid (ABA)-activated signal pathway, and transcription factors in TAM 111 and TAM 112

Since drought tolerance is closely related to genes response to heat stress^44^ and oxidative^5^, ABA-induced signal pathways^8^ and transcription factors^11^, comparison of dry–wet DEGs in these pathways between TAM 111 and TAM 112 was conducted according to 1855 genes with known functions. For response to heat stress, 73 genes showed different expression patterns under drought and wet conditions in the two cultivars with 41 being common, and six and 26 being specific in TAM 111 and TAM 112, respectively (Tables 4 and S8). Of the 41 common DEGs dry vs. wet, two genes with function of ATP binding and peroxidase activity had down-regulated expression, and the remained 39 up-regulated genes included 26 encoding heat-shock proteins and 13 had products of heat stress related proteins. Of the six dry–wet DEGs specific in TAM 111, one was down regulated and five were up regulated with two encoding transcription factors, two for heat shock proteins and one for transmembrane metal ion transporter. All dry–wet DEGs specific in TAM 112 were up regulated with 19 encoding heat shock proteins, six for transcription factors/co-factors and one for metal ion transporting (Tables 4 and S8).

**Table 4:** Genes related to response to water deprivation, heat and oxidative stresses, ABA-signaling and transcription factors in TAM 111 and TAM 112 revealed by gene expression comparison under dry and wet conditions at heading (HD) and grain filling (GF) stages.

Pathway/stage	Common in both cultivars		TAM 111-specific		TAM 112-specific	
	Down-regulated	Up-regulated	Down-regulated	Up-regulated	Down-regulated	Up-regulated
**Response to water deprivation stress (92 genes):**						
Only at HD	1	0	3	0	0	0
Only at GF	6	15	5	2	2	13
At both HD and GF	10	29	3	0	1	2
**Response to heat stress (73 genes):**						
Only at HD	0	0	0	0	0	17
Only at GF	0	3	1	5	0	6
At both HD and GF	2	36	0	0	0	3
**Response to oxidative stress (96 genes):**						
Only at HD	0	0	5	0	1	0
Only at GF	8	22	3	1	4	15
At both HD and GF	12	25	0	0	0	0
**ABA (ABA)-activated signal pathway (59 genes):**						
Only at HD	0	1	1	0	1	0
Only at GF	3	10	9	0	1	10
At both HD and GF	13	7	3	0	0	0
**Transcription factors (144 genes)**						
Only at HD	0	0	4	5	1	7
Only at GF	11	22	12	9	4	25
At both HD and GF	16	22	4	0	1	1

For genes with products involved in the processes of oxidation–reduction and adjusting cell redox homeostasis, 96 dry–wet DEGs were identified in TAM 111 and TAM 112 with 67 shown in both cultivars, and nine and 20 were specifically shown only in TAM 111 and TAM 112, respectively (Tables 4 and S9). Among those common oxidation–reduction genes in both cultivars, 20 were down regulated with 15 genes encoding oxidoreductases, three for peroxidase, one for hydrolase and one for zinc transporter. Of the 47 up-regulated common genes, 31 were encoding oxidoreductases, three for peroxidase, five for transferase, two for calcium binding, five for disulfide oxidoreductase and one for carboxylate reductase. Among dry–wet DEGs specific in cultivars, TAM 111 had one gene encoding transferase that was up-regulated and the other eight genes with seven encoding oxidoreductase and one for metal ion binding were all down-regulated. Of the 20 dry–wet DEGs specific in TAM 112, four genes encoding oxidoreductase and one for peroxidase were down regulated, and 15 genes with ten encoding oxidoreductase, two for electron transferase, one each for aldose reductase, disulfide oxidoreductase and calcium ion binding were up regulated (Tables 4 and S9).

A total of 59 genes were involved ABA-induced signal pathway that showed significant expression differences under dry and wet conditions in two cultivars with 34 dry–wet DEGs in common, and 13 and 12 being only shown in TAM 111 and TAM 112, respectively (Tables 4 and S10). Of the 34 common dry–wet DEGs, 16 genes were down-regulated with most having function of kinase activity and 18 genes being up-regulated with most related to biosynthesis, response to stresses of drought, cold, heat, and salt, and phosphatase related to stomatal lineage. The 13 TAM 111-specific DEGs were all down regulated and mostly related to stomatal movement and drought response; however, ten out of 12 TAM 112-specific DEGs were up-regulated with functions related to processes of carbohydrate metabolism, transmembrane transporting and stress responses at grain filling stage (Tables 4 and S10).

Expression comparison also detected 144 genes encoding transcription factors being differentially expressed under dry and wet conditions in TAM 111 and TAM 112 with 71 dry–wet DEGs being common in both cultivars and 34 and 39 being specific to TAM 111 and TAM 112, respectively (Tables 4 and S11). Of those common dry–wet DEGs, 44 genes that encoded transcription factors related to stress response of heat, salt and water deficit were up regulated. For dry–wet DEGs specifically appearing only in TAM 111 (34) or TAM 112 (39), 18 were down regulated in TAM 111 and six were down regulated in TAM 112. Of the 33 up-regulated transcription factor genes in TAM 112, 12 were directly involved in response to abiotic stresses (Table S11). Therefore, from gene expression in processes related to drought tolerance, the two cultivars had majority of dry–wet DEGs in common, and for DEGs dry vs. wet specific in each cultivar, TAM 112 had more genes than those of TAM 111 with the majority having increased expression under drought.

### Genes with unknown function in TAM 111 and TAM 112 and had differential expression under dry and wet conditions

A total of 399 genes with unknown functions were differentially expressed under dry and wet conditions in TAM 111 and TAM 112 with 258 encoding predicted uncharacterized proteins and 141 having no BLAST hits in DNA database NCBI and no corresponding GO terms can be found in protein databases. For genes encoding predicted uncharacterized proteins, 94 showed significant expression differences under dry and wet conditions in both cultivars with 44 being down regulated and 50 being up regulated. Among genes showed dry–wet expression difference only in one of the cultivars, TAM 111 had 72 genes with 33 being down regulated, 37 up regulated, and two being up regulated at heading but down regulated at grain filling. On the other hand, TAM 112 had 92 genes with 22 being down regulated and 70 being up regulated. Therefore, differential expression of these genes under dry and wet conditions detected in this study indicated that their encoded proteins may have functions involved in responding to drought.

For 141 gene sequences with no BLAST hits in NCBI DNA database and no similar GO terms matched in the protein databases, their sequence length ranged from 103 to 3311 bp with an average length of 542 bp (Table S12). Eight of them were differentially expressed in both TAM 111 and TAM 112 under dry and wet conditions at grain filling stage with five having no expression detected under drought (down regulated) and three being up regulated. Of the remaining 133 genes, 73 were differentially expressed only in TAM 111 and 60 only in TAM 112 under dry and wet conditions (Table S12). Of the TAM 111-specific no-hit genes, 41 were down regulated with 39 having no expression detected under drought, and 32 being up-regulated with 30 having no significant expression under well-watered conditions. Similarly, among 60 TAM 112-specific no-hit genes, 23 were down regulated with all of them having no significant expression under dry conditions, and 37 being up-regulated with 34 having no significant expression under well-watered conditions (Table S13). High sequence similarity of these genes with the corresponding DNA sequences in wheat reference genome indicated that all no BLAST-hit genes are very likely novel and involved in drought response in TAM 111 and TAM 112.

### Expression of homoeologous genes in TAM 111 and TAM 112 under dry and wet conditions

Of the 2254 genes differentially expressed in TAM 111 and TAM 112 under dry–wet condition at heading and grain filling stages, 231 were homoeologous genes with eight to 14 homoeologous genes from each homoeologous group (Table S14). All those homoeologous genes were differently expressed under dry–wet treatment in TAM 111 or TAM 112 and showed the same regulation pattern within each homoeologous group. Therefore, no compensated expressions were observed among homoeologous genes.

### RT-qPCR analysis

**Figure 6:**
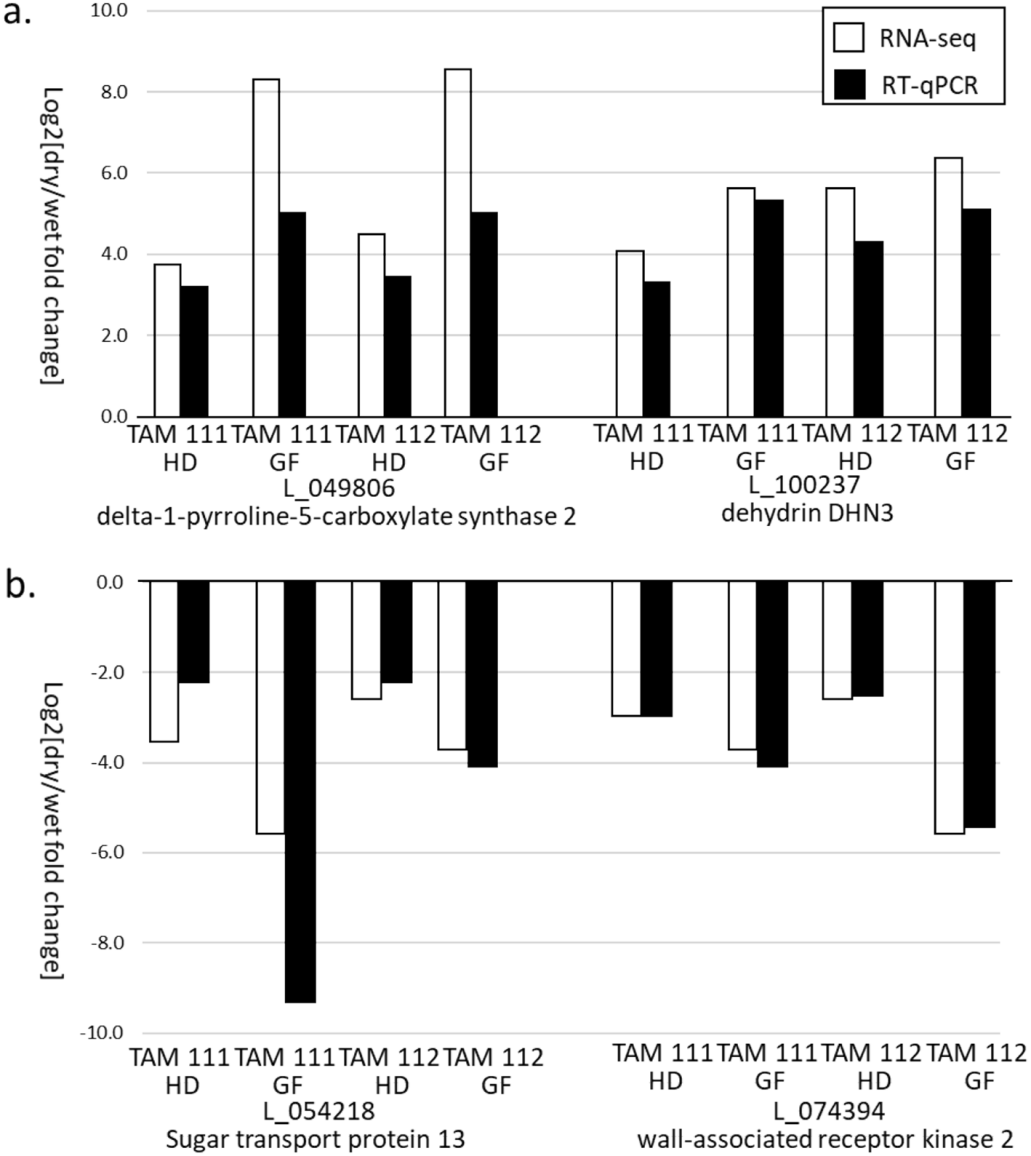
RT-qPCR validation of gene expression for two up regulated (**a**) and two down regulated (**b**) DEGs indicated through RNA-seq analysis. HD = heading stage, and GF = grain filling stage.

Four DEGs including two up regulated (L_049806 and L_100237 dehydrin DHN3) and two down regulated (L_054218 and L_074394) were selected for validating the expression level changes in dry and wet condition indicated through RNA-seq analysis. RT-qPCR using Actin gene as an endogenous control revealed similar gene expression fold change under different condition at heading and grain filling stages in both cultivars (Fig. 6). In addition, expression level change of five genes (L_057461 annotated as sucrose:fructan 6-fructosyltransferase, L_094799 annotated as Dehydrin 4, L_044222 annotated as Sucrose synthase 3, L_070049 annotated as NAC domain transcription factor and L_102286 annotated as Pyruvate dehydrogenase E1 α-2) have been validated through RT-qPCR in Reddy et al.^18^. Therefore, expression level change in nine genes were confirmed.

## Discussion

Drought stress at heading and grain filling stages of wheat can cause great reduction in grain yield, lower kernel weight and deteriorate end-use quality. Zampieri et al.^45^ estimated that the variability of 40% annual wheat productivity was mainly due to drought and heat stresses in the majority of wheat production regions. However, wheat tolerance to drought is a very complex trait regulated at different levels of gene expression, cellular response and physiological reaction with numerous minor effect genes involved^46^. Using transcriptomic analysis such as micro-array and RNA-seq to compare gene expression under drought and irrigated conditions provided a quick way of identifying genes related to drought response. Particularly, as DNA sequences of wheat whole genome are available now, RNA-seq can accurately identify genes and compare their expression levels under different conditions. In this study, we used RNA-seq and identified 2254 genes that are differentially expressed in cultivars TAM 111 and TAM 112 under dry and wet conditions at heading and grain filling stages, and RT-qPCR was used to validate expression of four DEGs in this study and five DEGs in Reddy et al.^18^. According to gene annotation through online DNA and protein databases, those differentially expressed genes were encoding key enzymes, transcription factors, signal receptors and cellular components that were involved in different biological processes and pathways for responding to abiotic stresses as well as defense responses. Particularly, genes identified in this study involved in pathways such as flavonoid biosynthesis, fructan biosynthesis in starch and sucrose metabolism and enzymes that having protein disulfide oxidoreductase activity and maintain cell redox homeostasis have already been reported to associate with drought tolerance in wheat^20,26,47^. Also, the subcellular locations of DEGs encoded proteins in peroxisome, nucleus and mitochondria associated with drought stress response were reported in many crops^48–50^. Therefore, drought response in TAM 111 and TAM 112 would have similar processes that drought signals promote plants to maintain water homeostasis and redox homeostasis to prevent acute cellular damage and membrane integrity^51–53^.

In this research, the differentially expressed genes encode aquaporin were all down-regulated for cellular osmotic adjustment to prevent water loss, which agreed with results from Reddy et al.^18^ and Zupin et al.^54^, and DEGs encoding heat shock protein, oxidoreductase, late embryogenesis abundant protein and dehydrin were mainly up-regulated in two cultivars under drought is in consistent with that increasing level of those proteins can maintain cellular redox homeostasis particularly at grain filling stage^55–62^. Expression of genes for transcription factor and proteins involved in ABA-induced signal pathway were either up- or down-regulated in both cultivars but were more up-regulated in TAM 112, which was in accordance with that ABA mostly plays the central role of regulating expression of genes for plant response to stress^11,63–65^, but different regulation in some genes between two cultivars indicated different strategies may be used for drought tolerance.

Winter wheat cultivars TAM 111 and TAM 112 were widely adapted to the Southern Great Plains and have excellent drought tolerance. Both TAM 111 and TAM 112 had 19.1% and 19.3% higher grain yield in dryland and 8.1% and 7.2% higher in irrigated field comparing with the grain yield of TAM 105^19^. From the same experiment, TAM 111 showed significant higher kernels per spike but TAM 112 showed significant higher thousand kernel weight than those of TAM 105 in both dryland and irrigated conditions. This is also consistent with what was found from more than 10 dryland and irrigated environments, TAM 112 had higher values of spikes per square meter, hardness and flour protein while TAM 111 had higher values of kernels per spike, single head grain weight, seed diameter and flour yield^66,67^. According to few studies about physiological and molecular basis of drought tolerance in the two cultivars, both had higher ABA content under drier conditions; however, TAM 112 had a higher ABA content and more robust expressions of genes involved in ABA-signal pathway compared to TAM 111 under dry condition^18^. Thapaet al.^68,69^ reported TAM 112 showed better water use efficiency than TAM 111 and thus had better tolerance under continuous drought conditions. Field yield trials also indicated that TAM 112 performed relatively better under sustained drought. In this research, out of 2254 dry–wet DEGs identified from the two cultivars, 1214 were common in both cultivars and 438 and 602 were present only in TAM 111 and TAM 112, respectively. Over 50% of dry–wet DEGs found in both cultivars supported that the two cultivars have robust drought tolerance derived from conserved regulatory network, whereas DEGs were found to be specific in each cultivar indicating that they have specific mechanisms against drought. Based on the hierarchical structure of biological processes, molecule functions and cellular components developed using genes with known functions, though two cultivars shared a large portion of dry–wet DEGs for each process, TAM 112 had more genes than TAM 111 that were up-regulated under drought. For processes such as protein phosphorylation, TAM 111 had more genes differentially expressed than TAM 112, but their expressions were mostly decreased under drought. Similar observations were found for genes with function of adenyl ribonucleotide binding or those with encoded proteins located in nucleus and plasma membrane with functions of regulating transcription and transmembrane transporting, which indicated that TAM 112 had relatively more active gene expression regulations than TAM 111 under drought.

Based on expression comparison for genes involved in responding to water deficit stress, heat stress, oxidative stress, ABA-activated signal pathway and transcription factors, TAM 112 always had a greater number of dry–wet DEGs than TAM 111 with significantly increased expression under dry conditions. This further suggests that active gene expression in TAM 112 support its better water use efficiency during drought periods, whereas TAM 111, though with most of dry–wet DEGs shared with TAM 112, had a greater number of down-regulated genes, potentially to protect it from drought and maintain its growth. Both greenhouse and field studies showed that TAM 112 had more tillers per spike and spikes m^-2^, lower stomatal conductance and gas exchange parameters, and higher photosynthetic water use efficiency under water deficit stress, and this was manifested in higher yield under drought^18,19,68^. On the other hand, higher yield of TAM 111 under drought was more associated with high spike and stem dry weight at anthesis and maturity^19^. In addition, TAM 111 tends to have more sterile spikelets per spike under water deficit stress compared to TAM112 although no significant difference was observed in their primary spikes^18^. Grain abortion and reduce plant cycle length were commonly known as strategies of plants response to long-term drought^51^. Therefore, the large portion of drought response genes shared by TAM 111 and TAM 112 contributed to their good drought tolerance. However, active expression of specific dry–wet DEGs in TAM 112 increased its resilience to and had higher grain yield under the sustained drought, whereas decreased expression of specific DEGs under drought may save more energy in TAM 111 for transporting carbohydrates from stems and spikes to seeds or even adjust gene expression to cause seed abortion in some spikelets to maintain grain production under drought.

Additionally, 399 genes showed differential expression in TAM 111 and TAM 112 have not been functionally annotated, which included 258 encoding predicted but uncharacterized proteins and 141 with no BLAST hits. Expression analysis of those genes in this research indicated they were related to drought stress responses. Since TAM 111 and TAM 112 are cultivars with unique drought tolerance mechanisms, those genes with unknown functions may be novel. Further research is necessary to reveal the protein products and functions of those genes.

Although many putative genes and QTLs conferring drought tolerance in wheat have been identified^46^, utilization of those genes/QTLs is challenging due to their small effects and instability caused by strong genetic × environment interaction^70^. Drought tolerance is a complex trait involving numerous genes involved in many biological processes, and selections of a few drought response genes with minor effects is most unlikely to confer a significant improvement on drought tolerance across diverse cultivars and environments^44^. Genomic selection of accumulating beneficial alleles at the whole genome level may provide a promising strategy for rapid and effective genetic gain on drought tolerance breeding^29,44^. Through RNA-seq, the sequences of the differentially expressed genes under drought conditions can be used to develop genic markers target to all drought response genes, and these markers can be combined with other markers covering the whole genome to conduct genomic selection toward drought tolerance improvement. The dry–wet DEGs identified in TAM 111 and TAM 112 from this research can be used to develop markers for efficiently utilizing the alleles of TAM 111 and TAM 112 for enhancing drought tolerance.

Lastly, this research used only flag leaf tissue for gene expression comparison at heading and grain filling stages, but root system of wheat plays very important roles for water intake from soil and affects water use efficiency. Comparing the expression of genes controlling root system architecture including root density, root length and root distribution^12^ of wheat thus need to be investigated. This research identified few genes related to root development though only flag leave tissue was used for expression analysis. In addition, gene expression for drought response at other stages such as stem elongation and early reproductive periods also plays important roles on grain yield^26^. Future research at other growth stages or utilizing root tissue to compare gene expression under dry–wet conditions will enrich our knowledge of drought tolerance mechanisms in wheat.

## Supplementary Information

Supplementary Information

## Data Availability

The RNA-seq reads used for this study are deposited at the National Center for Biotechnology Information Short Read Archive (https://www.ncbi.nlm.nih.gov/geo/query/acc.cgi?acc=GSE157033) under accession number GSE157033.
